# A case of gallbladder adenoma causing gallbladder haemorrhage

**DOI:** 10.1093/jscr/rjag328

**Published:** 2026-04-30

**Authors:** Takumi Yokoyama, Kenta Sui, Shintaro Okawa, Fumiaki Mukohara, Kazuma Iwata, Shimpei Tsudaka, Yuta Takahashi, Hiroki Otani, Norimitsu Tanaka, Masaaki Yano, Ryuichiro Ohashi

**Affiliations:** Department of Gastroenterological and General Surgery, Kagawa Prefectural Central Hospital, 1-2-1 Asahi-machi, Takamatsu, Kagawa 760-8557, Japan; Department of Gastroenterological and General Surgery, Kagawa Prefectural Central Hospital, 1-2-1 Asahi-machi, Takamatsu, Kagawa 760-8557, Japan; Department of Gastroenterological and General Surgery, Kagawa Prefectural Central Hospital, 1-2-1 Asahi-machi, Takamatsu, Kagawa 760-8557, Japan; Department of Gastroenterological and General Surgery, Kagawa Prefectural Central Hospital, 1-2-1 Asahi-machi, Takamatsu, Kagawa 760-8557, Japan; Department of Gastroenterological and General Surgery, Kagawa Prefectural Central Hospital, 1-2-1 Asahi-machi, Takamatsu, Kagawa 760-8557, Japan; Department of Gastroenterological and General Surgery, Kagawa Prefectural Central Hospital, 1-2-1 Asahi-machi, Takamatsu, Kagawa 760-8557, Japan; Department of Gastroenterological and General Surgery, Kagawa Prefectural Central Hospital, 1-2-1 Asahi-machi, Takamatsu, Kagawa 760-8557, Japan; Department of Gastroenterological and General Surgery, Kagawa Prefectural Central Hospital, 1-2-1 Asahi-machi, Takamatsu, Kagawa 760-8557, Japan; Department of Gastroenterological and General Surgery, Kagawa Prefectural Central Hospital, 1-2-1 Asahi-machi, Takamatsu, Kagawa 760-8557, Japan; Department of Gastroenterological and General Surgery, Kagawa Prefectural Central Hospital, 1-2-1 Asahi-machi, Takamatsu, Kagawa 760-8557, Japan; Department of Gastroenterological and General Surgery, Kagawa Prefectural Central Hospital, 1-2-1 Asahi-machi, Takamatsu, Kagawa 760-8557, Japan

**Keywords:** gallbladder adenoma, gallbladder haemorrhage, pyloric gland adenoma, cholecystectomy, case report

## Abstract

Gallbladder haemorrhage is a rare but life-threatening condition. We report a unique case of a 50-year-old female who presented with acute cholecystitis due to massive intracholecystic haemorrhage. A 17-mm pedunculated gallbladder polyp had been identified, but the patient opted for observation. Three weeks later, she presented to the emergency department with a chief complaint of epigastric pain. Computed tomography showed a distended gallbladder filled with hyperdense material, and magnetic resonance imaging confirmed a haematoma. Semi-urgent cholecystectomy was performed on the third hospital day. Pathological examination of the resected specimen revealed that a pyloric gland adenoma, which was positive for MUC6 and MUC5AC, had detached from its mucosal stalk, leading to significant hemorrhage. Although the primary indication for resecting large gallbladder polyps (≥10 mm) is malignancy risk, this case suggests that the risk of acute haemorrhagic complications from tumour detachment should also be recognized as a strong rationale for surgical intervention.

## Introduction

Gallbladder haemorrhage is a rare cause of gastrointestinal bleeding, often triggered by cholecystitis, trauma, or malignancy. Gallbladder adenomas are known as premalignant lesions; while they frequently require surgery due to carcinoma risk, reports of them causing acute hemorrhagic complications are rare. We report a unique case where a large pedunculated pyloric gland adenoma detached from its mucosal stalk, leading to massive intracholecystic hemorrhage and acute cholecystitis.

## Case report

**Figure 1:**
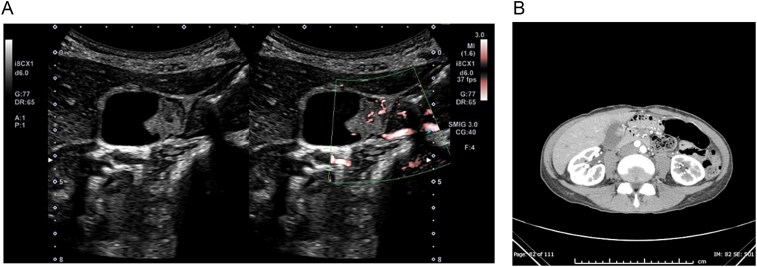
Initial imaging findings. (A) Abdominal ultrasound showing a 17-mm pedunculated polypoid lesion in the gallbladder body with internal blood flow signals. (B) Contrast-enhanced CT demonstrating a gallbladder polyp with enhancement (arrow). No signs of cholecystitis or hemorrhage were observed at this stage.

A 50-year-old woman was referred to our department with a 17-mm pedunculated gallbladder polyp incidentally discovered during a health checkup. Her past medical history was unremarkable. Laboratory tests showed a white blood cell count of 5.8x10^3^/μl (neutrophils 66.9%) and C-reactive protein of 0.02 mg/dl. Liver function tests and tumour markers (CEA: 2.5 ng/ml, CA19–9: 36 U/ml) were within normal ranges. Ultrasound and contrast-enhanced computed tomography (CT) revealed a 17-mm pedunculated polypoid lesion with blood flow signals in the gallbladder body (Fig. 1A and B). Surgical treatment was recommended, but the patient opted for observation.

**Figure 2:**
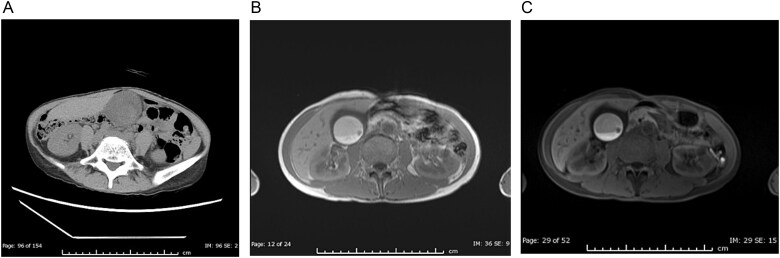
Imaging findings at emergency presentation. (A) Non-contrast CT showing a markedly distended gallbladder filled with high-density material (92 Hounsfield units), indicating acute intracholecystic hemorrhage. (B) T1-weighted MRI revealing high signal intensity within the gallbladder, consistent with a hematoma. The previously noted polypoid lesion is obscured by the haemorrhage. (C) T1-weighted MRI with fat suppression, the high-signal area within the gallbladder is not suppressed.

Three weeks later, she presented to our emergency department with acute epigastric pain. Physical examination revealed tenderness and a mobile mass in the abdominal midline, with no Murphy’s sign or peritoneal irritation. Due to a known contrast allergy, non-contrast CT was performed, showing a distended gallbladder with hyperdense material (CT value: 92 HU), suggesting hemorrhage. The gallbladder was deviated to the midline (Fig. 2A). Possible causes of the hemorrhage included bleeding due to gallbladder torsion (given the midline deviation) or bleeding from the previously identified gallbladder tumour. Since the patient’s vital signs were stable upon admission and the possibility of malignancy could not be entirely ruled out, we initially opted for conservative management to facilitate a thorough preoperative evaluation. During this period, magnetic resonance imaging (MRI) was performed, which showed high signal intensity on T1-weighted images within the gallbladder that was not suppressed on fat-suppression sequences, indicating the presence of a hematoma. No clear neoplastic lesion could be identified due to the hemorrhage (Fig. 2B and C). However, as the patient’s abdominal symptoms became refractory and persistent, we decided to perform a semi-urgent open cholecystectomy on the third day of hospitalization.

**Figure 3:**
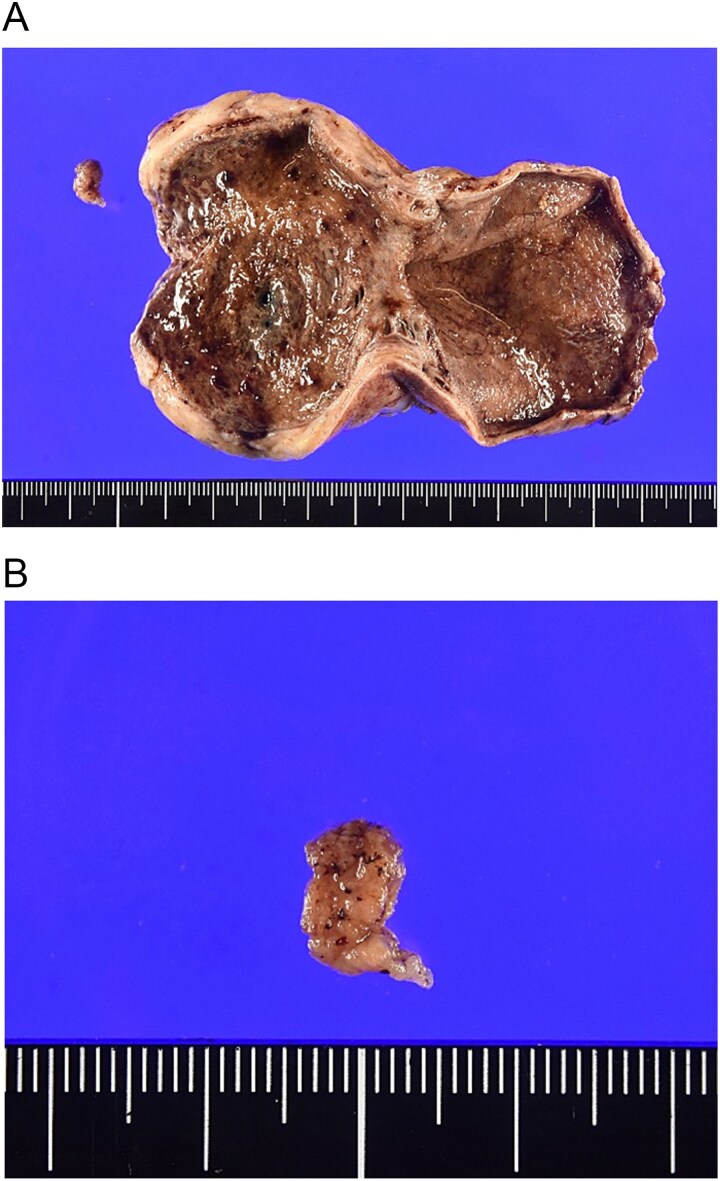
(A, B) Macroscopic findings. Resected specimen of the gallbladder. The lumen is filled with a large hematoma, and an 8-mm polypoid lesion (arrow) is found detached from the gallbladder wall.

**Figure 4:**
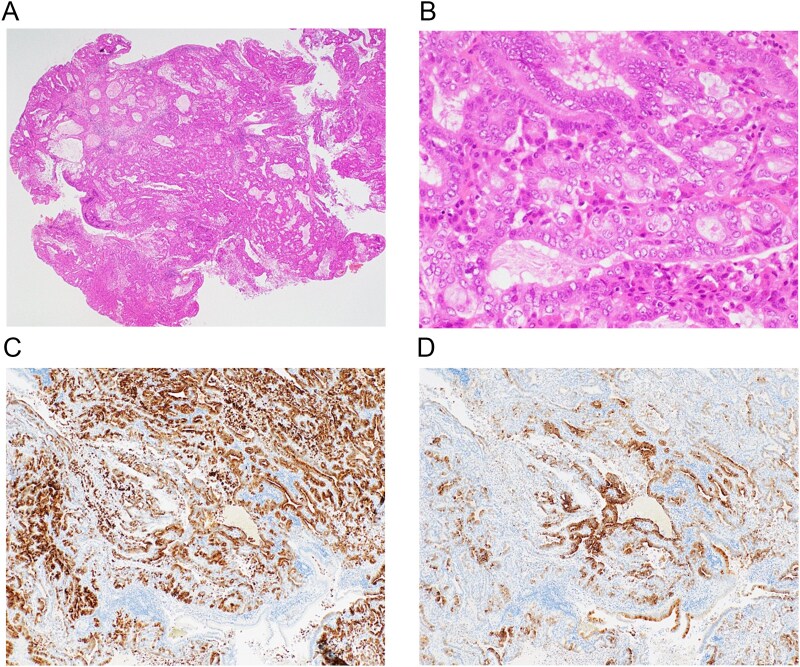
Histopathological findings. (A) Low-magnification view (Haematoxylin and eosin [H&E] staining) providing an overview of the polypoid lesion. (B) Higher-magnification view (H&E staining, ×40) revealing a pyloric gland adenoma with mild nuclear atypia and irregular glandular arrangement. There was no evidence of malignancy. (C) Immunohistochemical analysis showing that the tumour cells are diffusely positive for MUC6. (D) Immunohistochemical analysis showing that the tumour cells are partially positive for MUC5AC.

Open surgery was performed. The gallbladder was tense but adhered to the liver bed, with no torsion. Intraoperative incision of the specimen revealed the lumen filled with hematoma and an 8-mm adenoma that had clearly detached from its mucosal stalk (Fig. 3A and B). This size discrepancy between the initial imaging and the resected specimen could be attributed to several factors. First, the initial imaging may have measured the entire length of the pedunculated lesion, including its stalk. Second, the tumour likely underwent ischemic shrinkage or partial collapse after it detached from the mucosal stalk and lost its blood supply. Additionally, tissue shrinkage during the formalin fixation process may have contributed to the smaller pathological measurement. No gross signs of malignancy were observed; thus, liver bed resection and lymph node dissection were not performed. Histopathology showed dense proliferation of small glands with mild nuclear atypia (Fig. 4A and B). Immunohistochemistry was positive for MUC6 and partially for MUC5AC, leading to a diagnosis of pyloric gland adenoma (gastric type adenoma) (Fig. 4C and D). The patient was discharged on Day 6.

## Discussion

Hemorrhagic cholecystitis (gallbladder haemorrhage) is a rare disease, representing ~20% of biliary tract bleeding and 1% of upper gastrointestinal bleeding [1, 2]. Causes include gallstones, cholecystitis, tumours, ulcers, and vascular abnormalities such as pseudoaneurysms or atherosclerosis, trauma, and iatrogenic factors [3], with acute cholecystitis being the most common cause [4]. Reports also exist of cases associated with underlying conditions such as anticoagulant use [5, 6] and hemodialysis [7]. Two main mechanisms have been proposed for the development of acute cholecystitis with secondary hemorrhage: (i) Inflammation occurs first within the gallbladder due to gallstones or other causes, leading to mucosal degeneration, necrosis, and detachment, which results in the rupture of submucosal vessels and subsequent bleeding. (ii) Gallbladder haemorrhage occurs first due to arteriosclerosis of the gallbladder wall or gallbladder tumours; the haemorrhage itself or the formation of clots leads to increased intraluminal pressure, causing ischemic changes in the wall and secondary cholecystitis [7].

Regarding gallbladder polypoid lesions, definitions often used in Japan include ‘a localized elevated lesion on the mucosal surface distinguishable from the surrounding gallbladder mucosa’ [8, 9]. According to Horiguchi's classification [8], gallbladder polypoid lesions are classified into neoplastic and non-neoplastic lesions, and further into epithelial and non-epithelial lesions (Table 1). Adenomas, such as the one in this case, are classified as epithelial neoplastic lesions and are often reported as precancerous lesions. Regarding the frequency of cancer in gallbladder polypoid lesions, Tsuchiya et al. [10] reported that the frequency is 4.6% for lesions ≤5 mm and 9.3% for 6–10 mm, but rises sharply to 24.1% for 11–15 mm and 62.1% for 16–20 mm, indicating a significant increase when the tumour diameter exceeds 10 mm. Furthermore, regarding neoplastic lesions (cancer plus adenoma), it is reported that 43.1% of 11–15 mm lesions and 67.7% of 16–20 mm lesions are neoplastic, with the frequency becoming significantly higher when the size exceeds 10 mm [11, 12].

**Table 1:** Histological classification of gallbladder elevated lesions (proposed by Horiguchi [8])

I. Neoplastic lesion	II. Non-neoplastic lesion
1. Epithelial lesions	1. Epithelial lesions
i) Adenoma ii) Carcinoma in adenoma iii) Carcinoma	i) Hyperplastic polyp ii) Focal mucosal hyperplasia
2.Non-epithelial lesions	2. Non-epithelial lesions
	i) Cholesterol polyp ii) Inflammatory polyp iii) Granulation polyp iv) Adenomyomatous hyperplasia v) Heterotopia

According to current clinical guidelines, gallbladder polypoid lesions measuring 10 mm or more are generally indicated for surgical resection primarily due to their potential risk of malignancy [13]. In this case, the tumour diameter was 17 mm, which was considered an indication for surgery. However, due to the patient's preference, a policy of observation was adopted, and it is thought that during this course, the tumour detached, causing gallbladder haemorrhage and leading to acute cholecystitis. Generally, pedunculated polyps are considered to have a lower risk of malignancy compared to sessile lesions, which often leads to a more conservative management approach for such lesions. However, the unique clinical course observed in the present case—characterized by the detachment of a large pedunculated adenoma and subsequent massive hemorrhage—suggests that the risk of such acute complications should also be recognized as a valid rationale for surgical intervention. In this case, we believe that the large size and pedunculated nature of the adenoma contributed to its detachment, likely due to torsion of the mucosal stalk. Therefore, we propose that a large tumour size, even in pedunculated lesions where the risk of malignancy may be considered low, should be recognized as an important factor in recommending surgical resection to prevent rare but life-threatening events such as tumour-related hemorrhage.

## Data Availability

The datasets used during the current study are available from the corresponding author on reasonable request.
